# Microbiome multi-omics analysis reveals novel biomarkers and mechanisms linked with CD etiopathology

**DOI:** 10.1186/s40364-025-00802-1

**Published:** 2025-06-16

**Authors:** Gerard Serrano-Gómez, Francisca Yañez, Zaida Soler, Marc Pons-Tarin, Luis Mayorga, Claudia Herrera-deGuise, Natalia Borruel, Antonio Rodriguez-Sinovas, Marta Consegal, Isaac Manjón, Sara Vega-Abellaneda, Chaysavanh Manichanh

**Affiliations:** 1 Microbiome Lab, Vall d’Hebron Institut de Recerca (VHIR), 119-129 pg Vall d’Hebron, Barcelona, 08035 Spain; 2 Departament de Medicina, Universitat Autònoma de Barcelona, Barcelona, Spain; 3 Cardiovascular Diseases Research Group, Department of Cardiology, Vall d’Hebron Institut de Recerca (VHIR), Vall d’Hebron Barcelona Hospital Campus, Barcelona, Spain; 4 CIBER of Hepatic and Digestive Diseases (CIBERehd), Instituto de Salud Carlos III (ISCIII), Madrid, Spain

**Keywords:** Inflammatory bowel disease, Microbiome, Multi-omics, Adherent-invasive *Escherichia coli*, Virulence gene

## Abstract

**Background:**

The gut microbiome plays a key role in the development of inflammatory bowel disease (IBD), as imbalances in microbial composition are associated with immune dysfunction. However, the specific mechanisms by which certain microorganisms contribute to this process remain unclear.

**Methods:**

Here, we employed a multi-omics approach on fecal samples to identify novel microbiome markers and elucidate mechanisms underlying IBD. Shotgun metagenomics was applied to 212 samples (850 in total with validation cohort), shotgun metatranscriptomics to 103 samples and metabolomics to 105 samples. Machine learning techniques were used to predict disease and the three omics data were integrated to propose a mechanistic role of the microbiota.

**Results:**

Metagenomic analysis identified Crohn's disease (CD)-specific microbiome signatures, including a panel of 20 species that achieved a high diagnostic performance, with an area under the ROC curve (AUC) of 0.94 in an external validation set. Metatranscriptomic analysis revealed significant alterations in microbial fermentation pathways in CD, but not in ulcerative colitis (UC), highlighting disruptions that explain the depletion of butyrate—a key anti-inflammatory metabolite—observed in metabolomics analysis. Integrative multi-omics analyses further identified active virulence factor genes in CD, predominantly originating from the adherent-invasive *Escherichia coli* (AIEC). Notably, these findings unveiled novel mechanisms, including E. coli-mediated aspartate depletion and the utilization of propionate, which drives the expression of the ompA virulence gene, critical for bacterial adherence and invasion of the host’s macrophages. Interestingly, these microbiome alterations were absent in UC, underscoring distinct mechanisms of disease development between the two IBD subtypes.

**Conclusions:**

In conclusion, our study not only identifies promising novel biomarkers with strong diagnostic potential, which could be valuable in challenging clinical scenarios, but also offers an integrated multi-omics perspective on the microbial mechanisms underlying inflammation and virulence in Crohn's disease.

**Supplementary Information:**

The online version contains supplementary material available at 10.1186/s40364-025-00802-1.

## Background

Recent research on inflammatory bowel disease (IBD) has increasingly highlighted the crucial mechanistic role of the gut microbiome in the development and progression of the condition. Dysbiosis has been strongly implicated in triggering immune responses that exacerbate IBD symptoms, particularly through the actions of adherent-invasive *Escherichia coli* (AIEC) [1–3]. The discovery of AIEC was primarily based on culture techniques [4]. Dr. Arlette Darfeuille-Michaud and her team identified AIEC by isolating *E. coli* strains from ileal biopsies of Crohn's disease patients and culturing them under laboratory conditions. AIEC has been shown to invade the gastrointestinal tract and activate pro-inflammatory pathways, including Th1 and Th17 immune responses, which contribute to chronic inflammation [5, 6].

While it is well established that high levels of short-chain fatty acids (SCFAs), such as butyrate, in the gut lumen can induce anti-inflammatory effects [7, 8], recent targeted studies have shown that propionate, another SCFA, can enhance AIEC virulence under in vitro conditions [6, 9, 10]. Additionally, AIEC has demonstrated the metabolic potential to catabolize propionate in vivo, further implicating this SCFA in promoting AIEC's pathogenicity [11].

Although these findings are promising, determining whether these proposed mechanisms are occurring in vivo in IBD patients remains challenging, given that the results are derived from controlled in vitro studies. For this reason, multi-omics approaches—still underutilized in this field—could provide untargeted, comprehensive insights into the role of bacteria in the etiopathology of IBD.

In this study, we characterized the microbiome of patients with IBD and healthy controls using various omics technologies—specifically metagenomics, metatranscriptomics, and metabolomics—in an untargeted approach. This comprehensive analysis facilitated the identification of novel microbiome markers and mechanisms associated with the progression of IBD.

## Methods

### Study design

The study included a discovery cohort of 134 participants (34 patients with Crohn's disease (CD), 33 patients with ulcerative colitis (UC), and 67 healthy controls) in a longitudinal setting, previously described in Pascal et al. [12]. IBD patients were in remission at baseline and were followed up until they relapsed or for a maximum of 12 months if they remained in remission. Disease flare was assessed using the Harvey-Bradshaw Index (HBI) for CD patients and the Colitis Activity Index (CAI) for UC patients. In both cases, an individual was considered to be in remission when the HBI/CAI score was less than 4. Antibiotic intake within two months prior to the study was an exclusion criterion for all participants. Additional information regarding the patients'clinical data can be found in Supplementary Table 2.

For validation, we included 141 samples from 92 CD patients and 497 samples from healthy individuals across four cohorts (Supplementary Table 1). Cohort #1 comprised 12 newly diagnosed CD patients within the last six months, free of antibiotics for at least two months. Cohort #2 included 98 samples from 49 severe CD patients who had surgery due to therapy failure or complications, with samples collected before and six months post-surgery. Cohort #3 contained 31 CD patients free of antibiotics for two months. Cohort #4 involved 497 individuals aged 18 to 75 from Spain, free of antibiotics for three months and without diagnosed intestinal or autoimmune diseases. All cohorts were processed by our group using the same procedures as the main cohort.

### Data generation

Shotgun DNA metagenomes of 212 fecal samples from 134 participants were generated as previously described [13]. A frozen aliquot (200 mg) of each sample was suspended in 250 µl of 4 M guanidine thiocyanate, 500 µl of 5% N-lauroyl sarcosine, and 40 µl of 10% N-lauroyl sarcosine. Genomic DNA was extracted from the fecal samples, and shotgun metagenomic sequencing was performed on the 212 extracted genomic DNA samples using the Illumina HiSeq platform, yielding an average of 4.12 Gb per sample.

Shotgun metatranscriptomes from 103 samples collected from 55 participants (14 with CD, 14 with UC, and 27 HC) were generated. Total RNA extraction was performed following the protocol described by Cardona et al. [14]. Briefly, an aliquot (250 mg) of each sample was mixed with 500 µL of TE buffer, 0.8 g of zirconia/silica beads, 50 µL of a 10% SDS solution, 50 µL of sodium acetate, and 500 µL of acid-phenol. Physical disruption was achieved using a FastPrep apparatus (FP120, Thermo). After centrifugation of the lysate, nucleic acids were recovered from the aqueous phase and re-extracted with chloroform:isoamylalcohol. DNA was selectively digested, and RNA was purified using the RNeasy® Mini Kit (Cat. No. 74104, Qiagen), following the manufacturer’s instructions. Total RNA was subjected to an rRNA removal procedure using the Ribo-zero Magnetic kit according to the manufacturer’s instructions. Samples were then subjected to fragmentation of the remaining RNA molecules. Afterwards, complementary DNA (cDNA) of the RNA was synthesized following the same library preparation kit protocol. Each library was sequenced as paired-end 101-bp reads on the Illumina HiSeq 2000 platform (CNAG, Barcelona, Spain), yielding an average of 4.28 Gbp per sample.

Metabolomes from 105 samples collected from 55 participants (14 with CD, 14 with UC, and 27 HC) were generated as follows. A frozen aliquot (100 mg) of each sample was mixed with 1 mL of phosphate buffer (pH 7.4, 0.75 M) and vortexed for 2 min. Subsequently, 800 mg of previously sterilized 0.1 mm zirconia beads were added to the tubes, and mechanical disruption was performed for 3 to 5 min using a Beadbeater (Biospec Products ©). Samples were then centrifuged at 10,000 g for 1 min at 20 ºC. The resulting supernatant was filtered through a 0.2 μm membrane (Merck Millipore® SLMP025SS) and stored at −40ºC until subsequent NMR acquisition. To prepare for analysis, 500 μL of the filtrate was mixed with 100 μL of TSP (3-trimethylsilyl-2,2,3,3-tetradeuterosodium propionate) at a concentration of 1.16 mM in D2O (Deuterium Oxide, Sigma® 151,882 −25G), and the mixture was transferred to a 5 mm NMR tube for analysis (Cortecnet® 507-PP-8).

Samples were analyzed using a 400 MHz Bruker Advanced Spectrometer equipped with a cryoprobe and a 16-slot auto-sampler (Bruker, Barcelona, Spain). The sample temperature was maintained at 298 K, and the NoesyPr1d (90°-tl-90°-dmix-90°-FID) pre-saturation sequence was employed to suppress the residual water signal, utilizing low-power selective irradiation at the water frequency during the recycle delay (D1 = 5 s) and mixing time (D8 = 10 ms). Each spectrum's acquisition parameters included 256 scans of 21,826 complex data points with a spectral width of 12.015 ppm (acquisition time: 2.27 s). The resulting spectra for each sample were manually phased and baseline-corrected using the Chenomx NMR Suite (version 8.11). Metabolites were identified using the Chenomx 400 MHz Reference Compounds Library (version 11) and quantified with the same software, using TSP as the reference compound (shift at 0 ppm). This stool metabolite extraction protocol was optimized following the recommendations of several authors [15, 16]. A summary of the distribution of samples and individuals in each omics layer can be found in Supplementary Fig. 1.

### Metagenome and metatranscriptome sequence analysis

Metagenomic and metatranscriptomic sequencing reads were quality-controlled and decontaminated using KneadData v0.7.4 (https://huttenhower.sph.harvard.edu/kneaddata). Reads that mapped to the human genome and those with lengths below 50% of the total input read length were discarded. Taxonomic profiling of the metagenomic data was conducted using MetaPhlAn v4.0.3 [17], which employs single-copy marker genes to identify microbial species. The rel_ab_w_read_stats parameter was used to report the number of reads mapping to each identified clade. Functional profiling of the metagenomic and metatranscriptomic data was performed using HumanN v3.6 [18] with the UniRef90 database to identify reads that did not map to HumanN's custom pan-genome database.

To identify microbial virulence factors in both metagenomic and metatranscriptomic datasets, Bowtie2 v2.5.1 was employed to map the data to the DNA sequences of the core dataset from the Virulence Factor Database (VFDB) [19]. Reads that aligned to the reference sequence with less than 80% of their length or had a q-score below 30 were discarded. Unmapped reads were removed from the resulting gene table after CPM normalization and prior to further analyses.

### Statistical analysis

#### Metagenomics

The stratified taxonomic profiles generated by MetaPhlAn were collapsed to the species level before further analysis, resulting in a total of 1,381 identified species across the cohort. Due to the compositional nature of the samples and to account for unknown sampling fractions, differential abundance (DA) analysis of the microbial community was performed using ANCOM-BC [20]. The analysis tested for diagnosis (CD, UC, or HC) and included gender (female or male), BMI, age, and smoking habits (active smoker, ex-smoker, or non-smoker) as fixed effects while considering patient ID and time point as random effects. The resulting p-values were corrected for multiple testing using the false discovery rate (FDR). ANCOM-BC utilizes a linear regression framework that models raw absolute abundance data, estimates sampling fractions, and corrects for biases introduced by inter-sample variation while controlling FDR and maintaining statistical power.

Alpha diversity was estimated using the Chao1, Shannon, and Simpson indices to assess both richness and evenness within the bacterial community. Beta diversity was estimated using UniFrac distances. To quantify the degree of dysbiosis of IBD patients relative to healthy individuals, we calculated a dysbiosis score defined as the median weighted UniFrac distance of any given sample to a healthy plane built with the 67 HC samples. All comparisons of alpha diversity, beta diversity, and dysbiosis scores were performed using two-sided Mann–Whitney U tests.

#### Metatranscriptomics

MetaCyc pathways generated from the metatranscriptomics data by HumanN were filtered to remove unmapped and unintegrated reads. Pathways that did not reach an abundance threshold of 0.001 and a prevalence threshold of 0.1 (i.e., pathways representing less than 0.1% of the total sample abundance in at least 10% of the samples) were also removed, resulting in a total of 355 pathways. Since HumanN normalizes by read length in its pipeline and reports output in reads per kilobase (RPK), samples were sum-normalized to transcripts per million (TPM) and log2-transformed prior to further analysis. To avoid zero values, a pseudocount equal to the minimum non-zero value for each sample was added before log transformation. Differential expression analysis was performed using mixed-effects linear models, as implemented in the lme function of the R package nlme. The analysis tested for diagnosis (CD, UC, or HC) and included gender, age, and smoking habits as fixed effects, while patient ID and time point were treated as random effects. Differentially expressed pathway p-values were FDR corrected.

#### Metabolomics

A total of 23 different metabolites were identified and quantified at millimolar (mM) concentrations using Chenomx software. Differences in metabolite concentrations between disease conditions were assessed using the Mann–Whitney’s U test. Orthogonal Partial Least Squares Discriminant Analysis (OPLS-DA) was performed using the opls function from the ropls package in R [21]. Metabolite concentrations were mean-centered and scaled prior to building the model, which was validated by 100 random permutation tests and sevenfold cross-validation. Metabolite importance in the OPLS-DA was assessed using S-plots, which represent the covariance and correlation between the metabolites and the modeled class of the OPLS-DA. In this context, covariance quantifies the contribution of each metabolite to the model’s latent components, reflecting its influence on the model structure. In contrast, the correlation coefficient (p(corr)) captures the strength and direction of the linear association between each metabolite and the response variable. High covariance values indicate a substantial impact on component construction, while high p(corr) values denote strong predictive relevance with respect to the response.

Both models were trained using the caret package in R (package version 6.0–94), with the default number of trees (ntree = 500) and the optimal number of variables in each tree split was set to 2 (mtry = 2), which presented a better ROC. The models were trained using 2/3 of the discovery cohort as training set and 1/3 as a testing set using fivefold cross-validation. The resulting models were also tested with external validation cohorts.

#### Machine learning

Two random forest classifiers were trained to distinguish CD from healthy status using the bacterial signatures in the differential abundance analysis as predictors.

The first model included all the differentially abundant species, while the second model was limited to the top 10 enriched and top 10 depleted species between CD and HC. To identify the optimal number of predictors for the model, we performed stepwise ascending variable introduction (incorporating variables with the highest log2FC one at a time to the model and testing its performance). Although models with more than 20 predictors achieved higher performance metrics, many of these predictors exhibited very low prevalence (present in fewer than 25% of samples). To enhance generalizability and ensure practical applicability across diverse real-world scenarios, we constrained the final model to the top 20 predictors (10 enriched and 10 depleted) that balanced predictive power with broader prevalence across samples, thereby improving the robustness of the model.

Both models were trained using the caret package in R (package version 6.0–94), with the default number of trees (ntree = 500) and an optimized number of variables per split (mtry = 2), which yielded the best ROC. The models were trained on two-thirds of the discovery cohort using fivefold cross-validation, with the remaining one-third reserved for testing. The resulting models were also tested on external validation cohorts.

#### Virulence factor genes

Virulence factor gene tables generated by bowtie2 were sum-normalized to counts per million for DNA data and to transcripts per million for RNA data before removal of unmapped reads, resulting in a total of 622 virulence factor genes at DNA level and 373 virulence factor genes at RNA level. For RNA data, differential expression analysis was performed using mixed-effects linear models, as implemented in the lme function of the R package nlme. The analysis tested for diagnosis (CD, UC, or HC) and included gender, age, and smoking habits as fixed effects, while patient ID and time point were treated as random effects. Differentially expressed pathway p-values were FDR corrected.

## Results

### Cohorts and multi-omics data

The study included a discovery cohort of healthy controls (HC, *n* = 67 fecal samples), Crohn's disease (CD, *n* = 67), and ulcerative colitis (UC, *n* = 77) patients, along with three validation cohorts: HC (*n* = 497) and CD (*n* = 141) (Supplementary Table 1). Detailed patient characteristics for the discovery cohort are provided in Supplementary Table 2. All fecal samples underwent shotgun metagenomic analysis, with a subset also processed for metatranscriptomic and metabolomic analyses.

### Microbial signature for IBD and validation

**Fig. 1:**
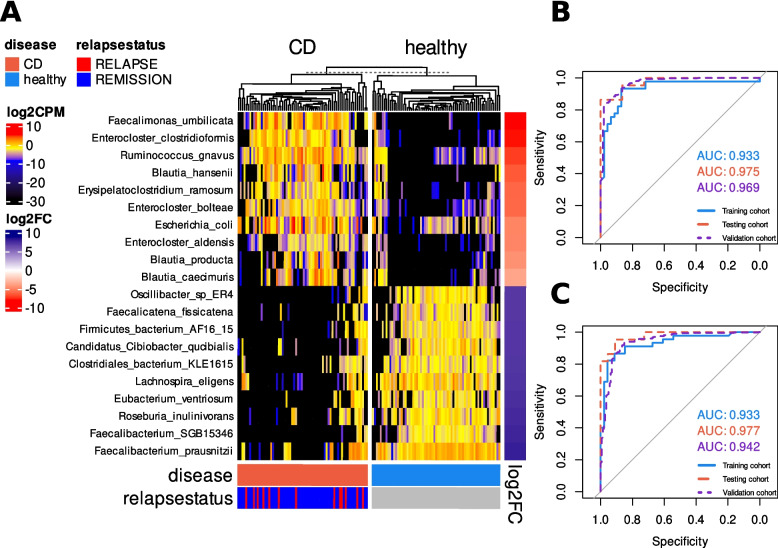
Microbial signatures for CD. Heatmap of the log2-transformed normalized abundance of the top 10 enriched and depleted species in CD (*n* = 68) patients compared to healthy controls (*n* = 67) (**A**). Performance (ROCAUC) of the random forest classifier built using the 174 DA species found (**B**) and a panel comprising the top 10 enriched and depleted species in CD (**C**). Differentially abundant species were obtained using ANCOM-BC

To identify potential microbial signatures in IBD, differential abundance (DA) analysis was conducted using ANCOM-BC and the taxonomic profiling of the 212 shotgun metagenomic fecal samples. One bacterial species, Clostridiaceae bacterium OM08-6BH (FDR = 0.0017), was found to be enriched in UC, while 156 species were enriched and 18 depleted in CD compared to HC (Fig. 1A, Supplementary Table 3). In contrast, 14 species were enriched and 101 depleted in CD compared to UC (Supplementary Fig. 2, Supplementary Table 4). *Faecalimonas umbilicata*, a species not previously associated with CD, was among the most significantly enriched species in CD (log2FC = −9.8, FDR = 5.8 × 10⁻^1^⁸). *Ruminococcus gnavus* and *Escherichia coli* were the most commonly reported enriched species, while *Faecalibacterium prausnitzii* was the most depleted in CD.

To evaluate the degree of dysbiosis in CD and UC, we calculated alpha-diversity indices (Chao1, Shannon, and Simpson), beta-diversity using UniFrac indices, and dysbiosis scores, defined as the median weighted UniFrac distance of any given sample to a healthy plane built with the 67 HC samples, based on the relative abundance of 1,381 microbial species identified from 212 shotgun metagenomic fecal samples. CD patients exhibited lower alpha-diversity (Shannon, Simpson & Chao1 *p* < 0.001 against HC and UC), greater inter-individual dissimilarity (PERMANOVA *p* < 0.001), and higher dysbiosis scores (p < 0.001 against HC and UC) compared to both HC and UC. In contrast, HC and UC were similar across all metrics (Supplementary Fig. 3).

We then used a machine learning classification framework to evaluate the predictive potential of the 174 differentially abundant species to distinguish CD from HC. The metagenomic cohort of 67 CD samples and 67 HC was split into a training and a test set, comprising two-thirds and one-third of the samples, respectively. A total of 141 CD samples and 497 HC, newly collected samples, were used for validation purposes; the information related to these samples is reported in the Supplementary Table 1. The random forest classifier was trained using the 174 species. The model demonstrated exceptional performance, achieving an AUC of 0.933 on the fivefold cross-validated training set, 0.975 on the test set, and 0.969 on the validation set (Fig. 1B).

To create a more focused model, we developed a second random forest classifier using only the 10 most enriched (*Faecalimonas umbilicata, Enterocloster clostridioformis, Ruminococcus gnavus, Blautia hansenii, Erysipelatoclostridium ramosum, Enterocloster bolteae, Escherichia coli, Enterocloster aldensis, Blautia producta and Blautia caecimuris*) and 10 most depleted (*Oscillibacter sp ER4, Faecalicatena fissicatena, Firmicutes bacterium AF16 15, Candidatus Cibiobacter qucibialis, Clostridiales bacterium KLE1615, Lachnospira eligens, Eubacterium ventriosum, Roseburia inulinivorans, Faecalibacterium SGB15346 and Faecalibacterium prausnitzii*) bacterial signatures in CD as predictors. This simplified model achieved an AUC of 0.933 on the fivefold cross-validated training set, 0.977 on the test set, and 0.942 on the validation set (Fig. 1C). Although the performance on the validation set was only slightly lower than the more complex model, this streamlined approach is significantly simpler. These results suggest that the microbial signatures highlighted in Fig. 1A captures the majority of the predictive power for distinguishing CD from HC, emphasizing their crucial role in CD pathophysiology.

### Metatranscriptome analysis reveals metabolic pathway shifts in CD

**Fig. 2:**
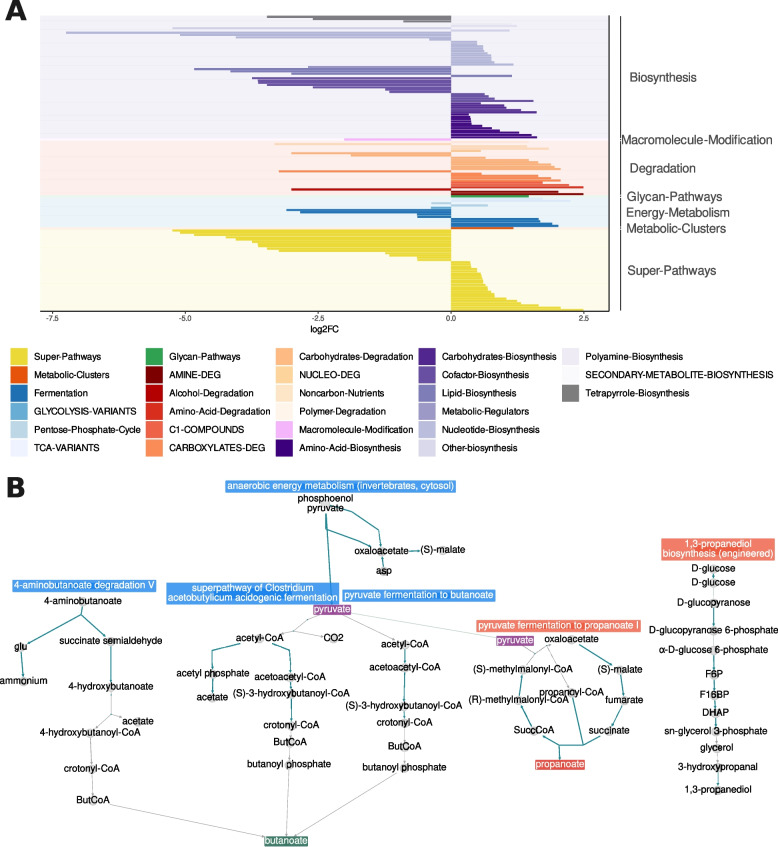
Pathway shifts in CD patients compared to HC. Differentially expressed pathways in the microbiome of CD patients (*n* = 27) compared to healthy controls (n = 49) using linear models (**A**). Pathways were grouped according to their level 1 MetaCyc classes (broader functionality) and colored by their level 2 MetaCyc classes (more specific). CD-depleted pathways are represented with negative log2FC, while CD-enriched pathways are positive. Differentially expressed fermentation between CD patients and healthy controls (**B**). In HC, fermentation pathways (blue) lead to the production of butyrate, while in CD (red), they lead to the production of propionate and 1,3-propanediol. Differentially expressed pathways were obtained using mixed-effects linear models

To evaluate IBD-associated changes in actively expressed microbial metabolic pathways, we performed a differential expression (DE) analysis using linear models on 355 MetaCyc pathways identified by HumanN3 from metatranscriptomic shotgun data of 103 fecal samples (HC, *n* = 49; CD, *n* = 27; UC, *n* = 27). No differentially expressed (DE) pathways were identified between UC and HC, while 50 pathways were significantly altered between CD and HC (FDR < 0.01, log2FC > 1), and 15 pathways showed differential expression between CD and UC (Supplementary Fig. 4). Significant shifts were observed in key pathways of the gut microbial ecosystem, including those involved in fermentation, carbohydrate and carboxylate degradation, and the synthesis of lipids, nucleotides, and cofactors (Fig. 2A). Notably, pathways related to carbohydrate and amino acid biosynthesis, amine degradation, and variants of the tricarboxylic acid (TCA) cycle were depleted in CD compared to HC (Fig. 2A). In contrast, pathways linked to pyruvate and butanoate catabolism, as well as *Faecalibacterium prausnitzii*, were enriched in HCs, consistent with previous findings on *F. prausnitzii*-derived butyrate production (Fig. 2B, Supplementary Fig. 5). Importantly, *F. prausnitzii* was the primary contributor to these pyruvate-producing pathways in HC, while in CD, pyruvate—likely sourced from other bacterial species—was utilized for propanoate production. A complete list of differentially expressed pathways, including their associated false discovery rates (FDR), log2 fold changes (log2FC), and full MetaCyc groups, can be found in Supplementary Table 5.

### Lower concentration of butyrate, aspartate, and glutamate in CD than in HC and UC

**Fig. 3:**
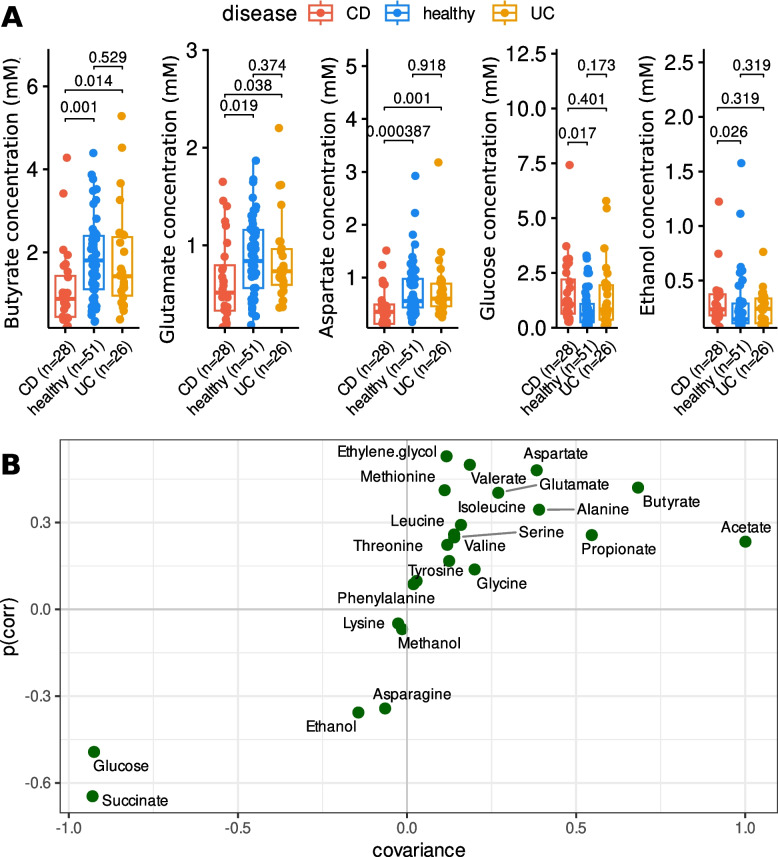
Differentially abundant metabolites in feces of IBD patients and HC. Enriched and depleted metabolites in stool samples of the participants, categorized by disease condition (CD *n* = 28, HC n = 51, UC *n* = 26) (**A**). S-plot of the OPLS-DA model between CD patients (*n* = 28) and healthy controls (*n* = 51) (sevenfold CV, 100 permutations, R2Y(cum) = 0.39, Q2Y(cum) = 0.19, pR2Y = 0.01, pQ2Y = 0.01) using metabolite concentration (**B**). The line in the boxplot depicts the median, the hinges depict the first and third quartile, and the whiskers depict values equal to the first quartile + 1.5 × IQR and the third quartile—1.5 × IQR. All individual points and p-values are shown. The S-plot represents both the covariance and correlation between the metabolites and the modeled class of the OPLS-DA model

Fecal metabolomic analysis of 105 samples (HC = 51, CD = 28, UC = 26) revealed that aspartate, butyrate, and glutamate were more abundant in HC, while ethanol and glucose were depleted compared to CD (Fig. 3A). No statistically significant differences were observed between UC and HC using the Mann–Whitney U test. To validate these findings, we conducted an orthogonal partial least squares discriminant analysis (OPLS-DA), which revealed a weak yet significant separation between CD and HC (Supplementary Fig. 6). The analysis highlighted the same top-contributing metabolites identified by the Mann–Whitney U test (Fig. 3B). Interestingly, succinate—a well-known pro-inflammatory metabolite [22–24]—which was not identified as a significant contributor in the previous method (*p* = 0.078), was now found to substantially contribute to CD status.

### Microbiome multi-omics analysis explains the depletion of aspartate in CD

To integrate the findings from the three omics datasets—metagenomics, metatranscriptomics, and metabolomics—we applied a similar approach to that described by Ning et al. [25]. Using the MetaCyc database, we linked the six previously identified metabolites to differentially expressed pathways. This analysis identified 18 pathways (Supplementary Table 6), which were then connected to their associated species, resulting in five pathways linked to both differentially abundant metabolites and species (Supplementary Table 7). Remarkably, all of these pathways were associated with *Escherichia coli*, with only one pathway linked to another species, *Klebsiella pneumoniae*. Notably, among the *E. coli*-related pathways, the most overexpressed in CD were those involved in the consumption of aspartate—a metabolite known to attenuate intestinal injury and enhance gut integrity [26, 27]—for the synthesis of nucleotides, purines, pyrimidines, and histidine.

### Microbial virulence factors are enriched in CD patients at both DNA & RNA level

**Fig. 4:**
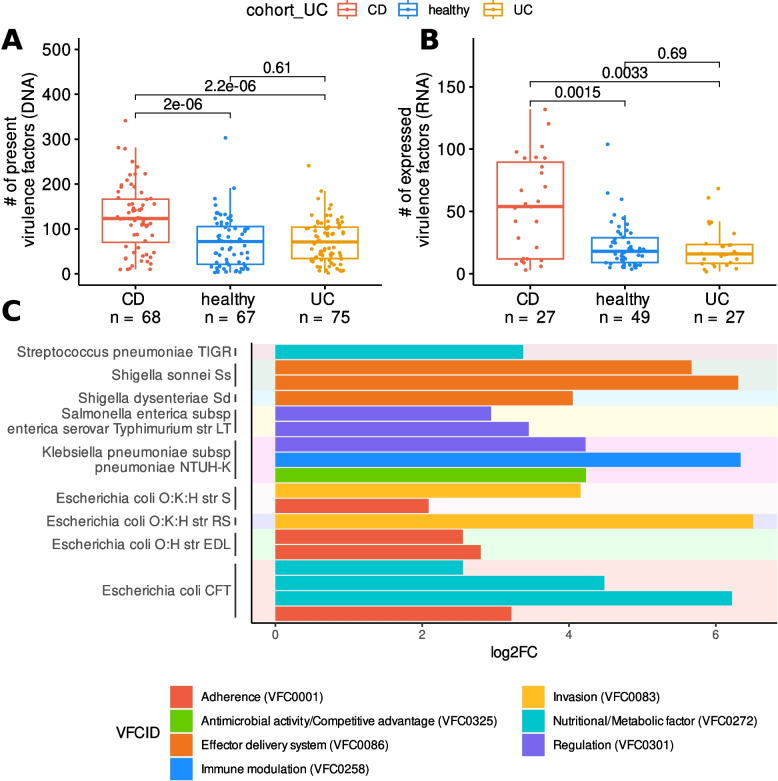
Study of virulence factor genes. Comparison of virulence factor (VF) genes detected at DNA level (CD *n* = 68, HC *n* = 67, UC *n* = 77) (**A**) and actively transcribed at RNA level (CD *n* = 27, HC *n* = 49, UC *n* = 27) (**B**) depending on disease condition. Representation of CD-enriched actively transcribed virulence factor genes compared to healthy controls (**C**). VF genes were grouped by microorganisms of provenance and coloured by their function, according to the Virulence Factor Database (VFDB). The line in the boxplot depicts the median, the hinges depict the first and third quartile, and the whiskers depict values equal to the first quartile + 1.5 × IQR and the third quartile—1.5 × IQR. All individual points and *p*-values are shown

To investigate the impact of microbial virulence factors in IBD, we mapped the metagenomic and metatranscriptomic datasets to the Virulence Factor Database (VFDB). Patients with CD exhibited a significantly higher expression of virulence factors compared to HC and UC (Fig. 4AB). Differential expression analysis between CD and HC identified a total of 18 RNA-expressed virulence factors that were enriched in CD. Among these, nine were derived from various strains of *E. coli*, three from *Shigella spp*., three from *Klebsiella pneumoniae*, two from *Salmonella enterica*, and one from *Streptococcus pneumoniae* (Fig. 4C). Notably, six of the nine *E. co*li pathways were associated with adherence or invasion, underscoring the critical role of AIEC in CD.

### AIEC virulence factor ompA is related to propionate and its metabolism

**Fig. 5:**
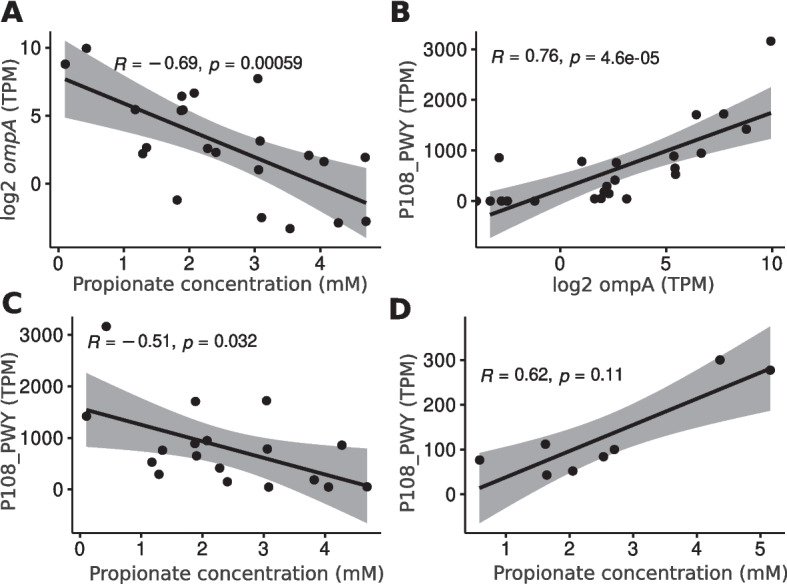
Correlation between metabolites, pathways and virulence factor genes. Spearman’s correlation between ompA and propionate (**A**) in CD, between P108-PWY and propionate in CD (**B**), and between P108-PWY and ompA in both CD (**C**) and healthy controls (**D**) (CD *n* = 25, HC *n* = 23, UC *n* = 22). All individual points, p-values, and correlation coefficients are shown

We further explored the role of virulence factors (VFs), focusing on *ompA*, the most differentially expressed and prevalent VF in CD and HC (Supplementary Fig. 7). To investigate this, we analyzed the correlation between *ompA* expression and the concentrations of all detected metabolites. This analysis revealed a negative correlation between *ompA* and propionate (R = −0.69, FDR = 0.01) in CD patients expressing o*mpA *(Fig. 5A). In contrast, propionate did not show significant correlation with o*mpA* expression in HC after false discovery rate (FDR) correction.

The expression of *ompA* was positively correlated with the P108-PWY pathway in both CD and HC, which actively produces propionate (Fig. 5B, C). However, in CD patients, propionate concentration was negatively correlated with P108-PWY expression (R = −0.51, *p* = 0.03) (Fig. 5D), while a positive correlation trend was observed in HC (R = 0.62, *p* = 0.11). These findings suggest that propionate produced by this pathway does not accumulate in CD patients, whereas it tends to do so in healthy individuals.

## Discussion

These findings highlight significant disruptions in the gut microbiota of CD patients, with reduced bacterial richness and diversity compared to both HC and UC patients. The reduced alpha diversity (richness and evenness) and distinct beta diversity (overall community composition) suggest that the gut microbiota is more profoundly affected in CD than in UC. This supports the idea that microbial dysbiosis is more severe and specific in CD, potentially playing a larger role in its pathology. Only one differentially abundant species (Clostridiaceae bacterium OM08-6BH) was identified in UC patients compared to HC, implying that UC's gut microbial changes are subtler than those in CD, and involve other microbial compartments such as fungi, viruses or even plasmids. Further investigation is required to better understand the pathogenesis of UC. In contrast, CD patients showed substantial disruptions, characterized by the enrichment of harmful species such as *Escherichia coli* and *Ruminococcus gnavus*, and the depletion of beneficial butyrate-producing bacteria like *Faecalibacterium prausnitzii*. These results align with previous studies [12, 28, 29], but the identification of *Faecalimonas umbilicata* as a novel, highly enriched species in CD introduces a new potential biomarker for the disease. Current evidence indicates that *F. umbilicata* is significantly enriched in individuals with IBD compared to healthy controls. A recent metagenomic analysis identified *F. umbilicata* as one of the few species-level taxa consistently elevated in IBD cohorts, suggesting a potential association with disease status [30]. However, the its functional role in gut inflammation remains unclear. Although its increased abundance in IBD patients implies a possible link, there is currently no direct evidence that *F. umbilicata* exerts pro-inflammatory effects or contributes causally to IBD pathogenesis. Further research, particularly functional studies, is needed to clarify its role in gut inflammation.

The construction of a highly accurate random forest model using just 20 bacterial species as predictors highlights the potential of gut microbiome profiling as a diagnostic tool. The model's strong performance, validated on a large independent cohort, suggests that microbiome data could be a powerful, non-invasive method to aid in diagnosing CD. While endoscopy remains the gold standard for diagnosis, the microbiome's predictive accuracy, combined with its cost-effectiveness and speed, could make it a valuable complementary approach, especially in ambiguous clinical cases where endoscopy alone might lead to misdiagnosis (which occurs in up to 10% of IBD patients). However, it is important to acknowledge that Cohort #2 included post-surgical CD patients, whose gut microbiota may have been influenced by long-term alterations following intestinal resection or perioperative antibiotic use. These factors may affect the generalizability of our findings, particularly in surgery-naive populations, and should be considered when interpreting the diagnostic potential of the model. Overall, these findings underscore the importance of gut microbiota in CD pathology and suggest a promising role for microbiome-based diagnostics in clinical practice.

Butyrate, a key metabolite for maintaining gastrointestinal homeostasis, is often depleted in individuals with IBD, along with butyrate-producing bacteria [31]. Previous studies have demonstrated that dietary supplementation with aspartate improved intestinal function and reduced overall intestinal damage by modulating the host’s inflammatory response in the intestines of LPS-challenged piglets [26, 27]. Our findings suggest that depletion of butyrate and aspartate may disrupt GI homeostasis in CD.

Previous metatranscriptomic studies in IBD revealed increased microbial transcriptional activity [32], but shifts in microbial metabolic functions at the pathway level were not examined. Our study characterized actively transcribed pathways and identified broad metabolic alterations in CD patients. In healthy individuals, pyruvate is fermented to butyrate by the microbiota, but in CD patients, dysbiotic microbiota ferment pyruvate into propionate and 1,3-propanediol, potentially triggering inflammation. This is supported by recent research linking propionate metabolism to inflammatory responses via Th17 cells and IL-1β [6].

**Fig. 6:**
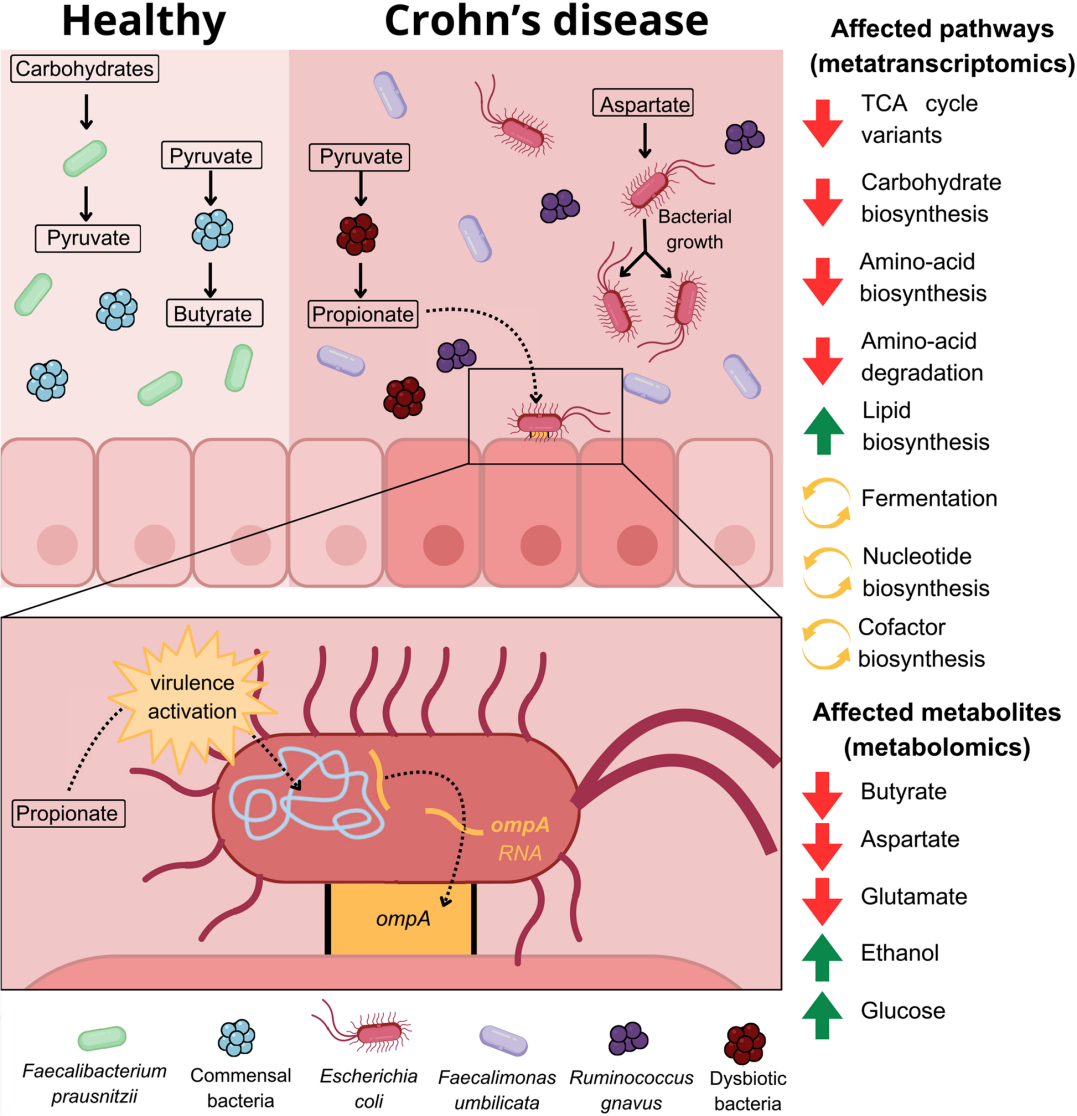
Bacterial dysbiosis drives profound changes in the gut environment of CD patients. These changes include the displacement of commensal bacteria, predominantly *F. prausnitzii* in healthy individuals, by dysbiotic bacterial communities dominated by *F. umbilicata* in CD patients. Additionally, microbial metabolic pathway activity is altered, leading to shifts in metabolite concentrations within the GI tract of affected individuals. Notably, integrative analysis of metagenomics, metatranscriptomics, and metabolomics revealed that *E. coli* utilizes aspartate to promote its growth and consumes propionate to express virulence factor genes such as ompA, which is involved in host adherence and invasion

Our multi-omic analysis identified an increased number of virulence factor genes in CD patients at both DNA and RNA levels. Differential expression analysis highlighted the importance of adherence and invasion genes from *E*. *coli*, underscoring the role of AIEC in CD. Expression of the *ompA*, the most overexpressed virulence factor in CD, was negatively correlated with propionate concentration. Although propionate promotes AIEC virulence in animal and in vitro models [6, 9], our data suggest that in CD patients, propionate is being utilized for virulence factor expression and does not accumulate. The positive correlation between the P108-PWY pathway and ompA in both CD and HC, alongside the negative correlation between the pathway and propionate in CD, indicates that propionate in CD may be used for virulence factor expression, aligning with previous findings on *E. coli's* capacity to produce more ompA in the presence of propionate [10]. We summarized all the previously discussed findings in Fig. 6. This targeted multi-omics integration allowed us to trace how microbial taxa (metagenomics) and their actively expressed genes (metatranscriptomics) were functionally associated with shifts in metabolite concentrations (metabolomics). For instance, while the virulence-associated gene ompA was identified through metatranscriptomic data, it was highly expressed in CD patients and linked to *E. coli*, a species also indirectly detected at the transcriptomic level. Notably, ompA expression correlated with reduced levels of propionate, suggesting that this short-chain fatty acid may be actively consumed during the expression of virulence factors. This pattern provides a mechanistic link between metabolic alterations and bacterial behavior. By combining the three omics layers, we were able to strengthen causal inferences and construct a more coherent model of bacterial dysbiosis and inflammation in CD.

Our multi-omics approach has shed light on microbiome mechanisms in IBD, particularly in CD, but there are some limitations. The integration of three different omics technologies made external validation challenging, as comparable multi-omics studies in IBD are virtually non-existent. Additionally, while NMR enables metabolite quantification using a reference compound, its lower sensitivity compared to mass spectrometry may have limited our ability to detect further metabolic differences in IBD patients.

Moreover, many of our findings centered on *E. coli*, specifically AIEC, which is likely involved in CD etiology. However, given *E. coli’s* extensive characterization and strong database representation, it is possible that other critical microbial factors are being overlooked due to the bias in available microbial knowledge.

Despite these limitations, our study successfully identified significant taxonomic and metabolic shifts in the gut microbiome of CD patients, linking these changes to AIEC pathogenicity using patient-derived samples. This represents an important advancement in understanding microbiome dynamics in IBD from a comprehensive perspective. Additionally, our findings provide a strong foundation for future experimental studies to validate these results and further investigate the CD-associated mechanisms uncovered in this research. Finally, we emphasize that the greater degree of bacterial dysbiosis in CD compared to UC has not been widely recognized until now. Distinguishing the biomarkers and mechanisms underlying the pathophysiology of these two IBD subtypes is crucial, as each may require distinct microbiota-based therapeutic strategies and diagnostic tools.

## Supplementary Information

Additional file 1: Supplementary Figures 1-7.

Additional file 2: Supplementary Tables 1-7.

## Data Availability

Shotgun metagenomic data of the discovery cohort analysed during the current study are available in the in the NCBI database under the following Bioproject accession number: PRJNA1171764. Metatranscriptomics and metabolomics data of the discovery cohort and shotgun metagenomic data of the validation cohorts are available from the corresponding author on reasonable request.
