# Supplementary material for: Microbiome multi-omics analysis reveals novel biomarkers and mechanisms linked with CD etiopathology
Source: Biomark Res. 2025 Jun 16;13:85. doi: 10.1186/s40364-025-00802-1 (PMC12172205; doi:10.1186/s40364-025-00802-1)
Supplement: Supplementary file 1 — Additional file 1: Supplementary Figures 1-7. [file 40364_2025_802_MOESM1_ESM.pdf]

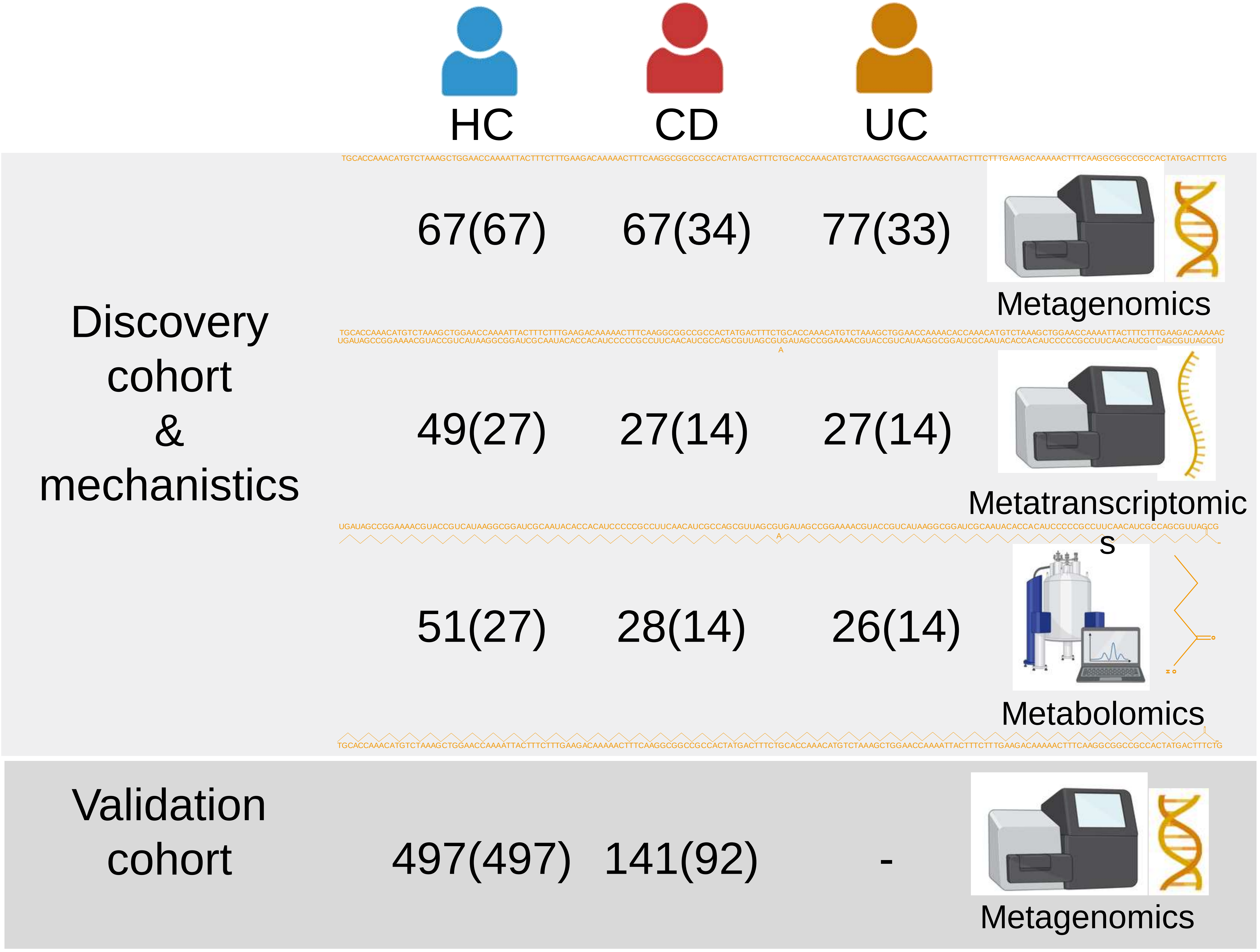
HC
CD
UC
67(67)
67(34)
77(33)
Metagenomics
Discovery cohort & mechanistics
49(27)
27(14)
27(14)
Metatranscriptomics
51(27)
28(14)
26(14)
Metabolomics
Validation cohort
497(497)
141(92)
-
Metagenomics

Supplementary Figure 1. Summary of the study cohorts. Numbers indicate the number of samples & number of individuals in parenthesis.

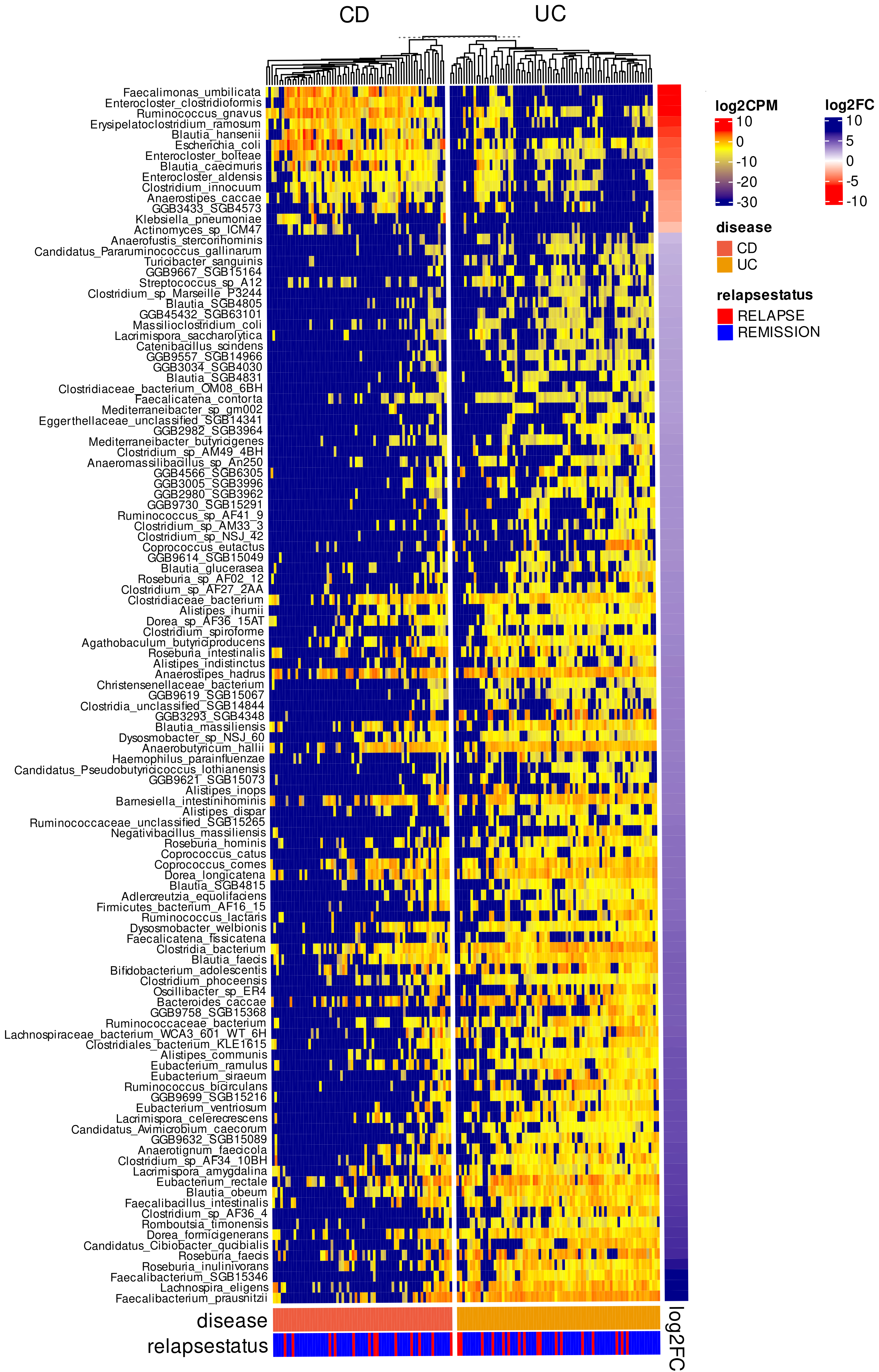

CD
UC
Faecalimonas_umbilicata
Enterocloster_clostridioformis
Ruminococcus_gnavus
Erysipelatoclostridium_ramosum
Blautia_hansenii
Escherichia_coli
Enterocloster_bolteae
Blautia_caecimuris
Enterocloster_aldensis
Clostridium_innocuum
Anaerostipes_caccae
GGB3433_SGB4573
Klebsiella_pneumoniae
Actinomyces_sp_ICM47
Anaerofustis_stercorihominis
Candidatus_Pararuminococcus_gallinarum
Turicibacter_sanguinis
GGB9667_SGB15164
Streptococcus_sp_A12
Clostridium_sp_Marseille_P3244
Blautia_SGB4805
GGB45432_SGB63101
Massilioclostridium_coli
Lacrimispora_saccharolytica
Catenibacillus_scindens
GGB9557_SGB14966
GGB3034_SGB4030
Blautia_SGB4831
Clostridiaceae_bacterium_OM08_6BH
Faecalicatena_contorta
Mediterraneibacter_sp_gm002
Eggerthellaceae_unclassified_SGB14341
GGB2982_SGB3964
Mediterraneibacter_butyricigenes
Clostridium_sp_AM49_4BH
Anaeromassilibacillus_sp_An250
GGB4566_SGB6305
GGB3005_SGB3996
GGB2980_SGB3962
GGB9730_SGB15291
Ruminococcus_sp_AF41_9
Clostridium_sp_AM33_3
Clostridium_sp_NSJ_42
Coprococcus_eutactus
GGB9614_SGB15049
Blautia_glucerasea
Roseburia_sp_AF02_12
Clostridium_sp_AF27_2AA
Clostridiaceae_bacterium
Alistipes_ihumii
Dorea_sp_AF36_15AT
Clostridium_spiroforme
Agathobaculum_butyriciproducens
Roseburia_intestinalis
Alistipes_indistinctus
Anaerostipes_hadrus
Christensenellaceae_bacterium
GGB9619_SGB15067
Clostridia_unclassified_SGB14844
GGB3293_SGB4348
Blautia_massiliensis
Dysosmobacter_sp_NSJ_60
Anaerobutyricum_hallii
Haemophilus_parainfluenzae
Candidatus_Pseudobutyricicoccus_lothianensis
GGB9621_SGB15073
Alistipes_inops
Barnesiella_intestinihominis
Alistipes_dispar
Ruminococcaceae_unclassified_SGB15265
Negativibacillus_massiliensis
Roseburia_hominis
Coprococcus_catus
Coprococcus_comes
Dorea_longicatena
Blautia_SGB4815
Adlercreutzia_equolifaciens
Firmicutes_bacterium_AF16_15
Ruminococcus_lactaris
Dysosmobacter_welbionis
Faecalicatena_fissicatena
Clostridia_bacterium
Blautia_faecis
Bifidobacterium_adolescentis
Clostridium_phoceensis
Oscillibacter_sp_ER4
Bacteroides_caccae
GGB9758_SGB15368
Ruminococcaceae_bacterium
Lachnospiraceae_bacterium_WCA3_601_WT_6H
Clostridiales_bacterium_KLE1615
Alistipes_communis
Eubacterium_ramulus
Eubacterium_siraeum
Ruminococcus_bicirculans
GGB9699_SGB15216
Eubacterium_ventriosum
Lacrimispora_celerecrescens
Candidatus_Avimicrobium_caecorum
GGB9632_SGB15089
Anaerotignum_faecicola
Clostridium_sp_AF34_10BH
Lacrimispora_amygdalina
Eubacterium_rectale
Blautia_obeum
Faecalibacillus_intestinalis
Clostridium_sp_AF36_4
Romboutsia_timonensis
Dorea_formicigenerans
Candidatus_Cibiobacter_qucibialis
Roseburia_faecis
Roseburia_inulinivorans
Faecalibacterium_SGB15346
Lachnospira_eligens
Faecalibacterium_prausnitzii
disease
relapsestatus
log2FC
log2CPM
10
0
-10
-20
-30
log2FC
10
5
0
-5
-10
disease
CD
UC
relapsestatus
RELAPSE
REMISSION

Supplementary Figure 2. Heatmap of the log2-transformed normalized abundance of the enriched and depleted species in CD patients (n=67) compared to UC patients (n=77). Differentially abundant species were obtained using ANCOM-BC.

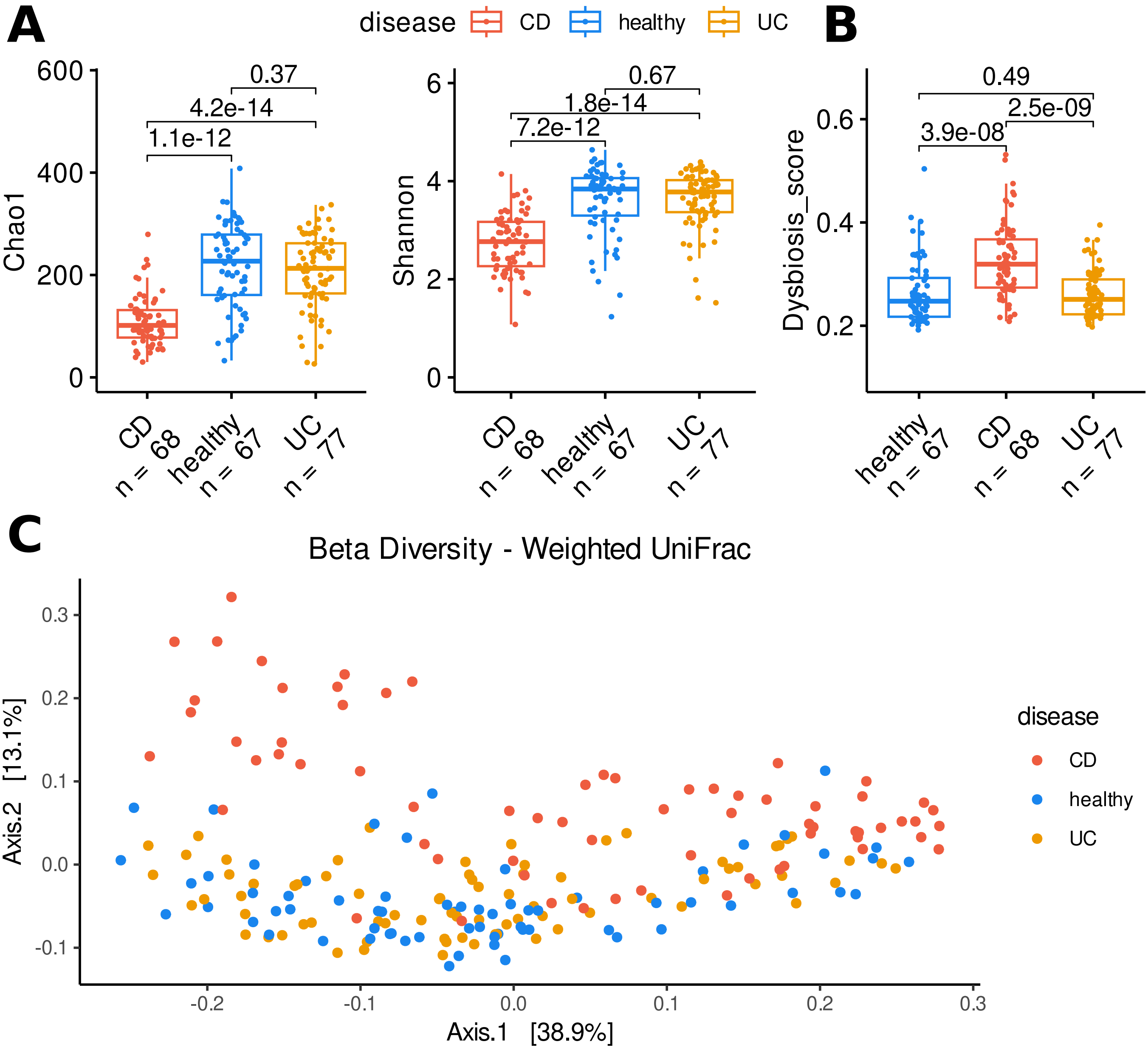
A
B
C
disease
CD
healthy
UC
Chao1
Shannon
Dysbiosis_score
0.37
4.2e-14
1.1e-12
0.67
1.8e-14
7.2e-12
0.49
2.5e-09
3.9e-08
CD n = 68
healthy n = 67
UC n = 77
Beta Diversity - Weighted UniFrac
Axis.1 [38.9%]
Axis.2 [13.1%]

Supplementary Figure 3. Alpha, beta diversity, and dysbiosis score based on the taxonomic profile of DNA samples (CD n=68, HC n=67, UC n=77). For alpha diversity, the richness of bacterial species was assessed using the Chao1 index (A), while for evenness, the Shannon index (B) was used. Weighted UniFrac distance was used for dysbiosis score (B) and beta diversity (C). The line in the boxplot depicts the median, the hinges depict the first and third quartile, and the whiskers depict values equal to the first quartile + 1.5 x IQR and the third quartile - 1.5 x IQR. All individual points and p-values are shown.

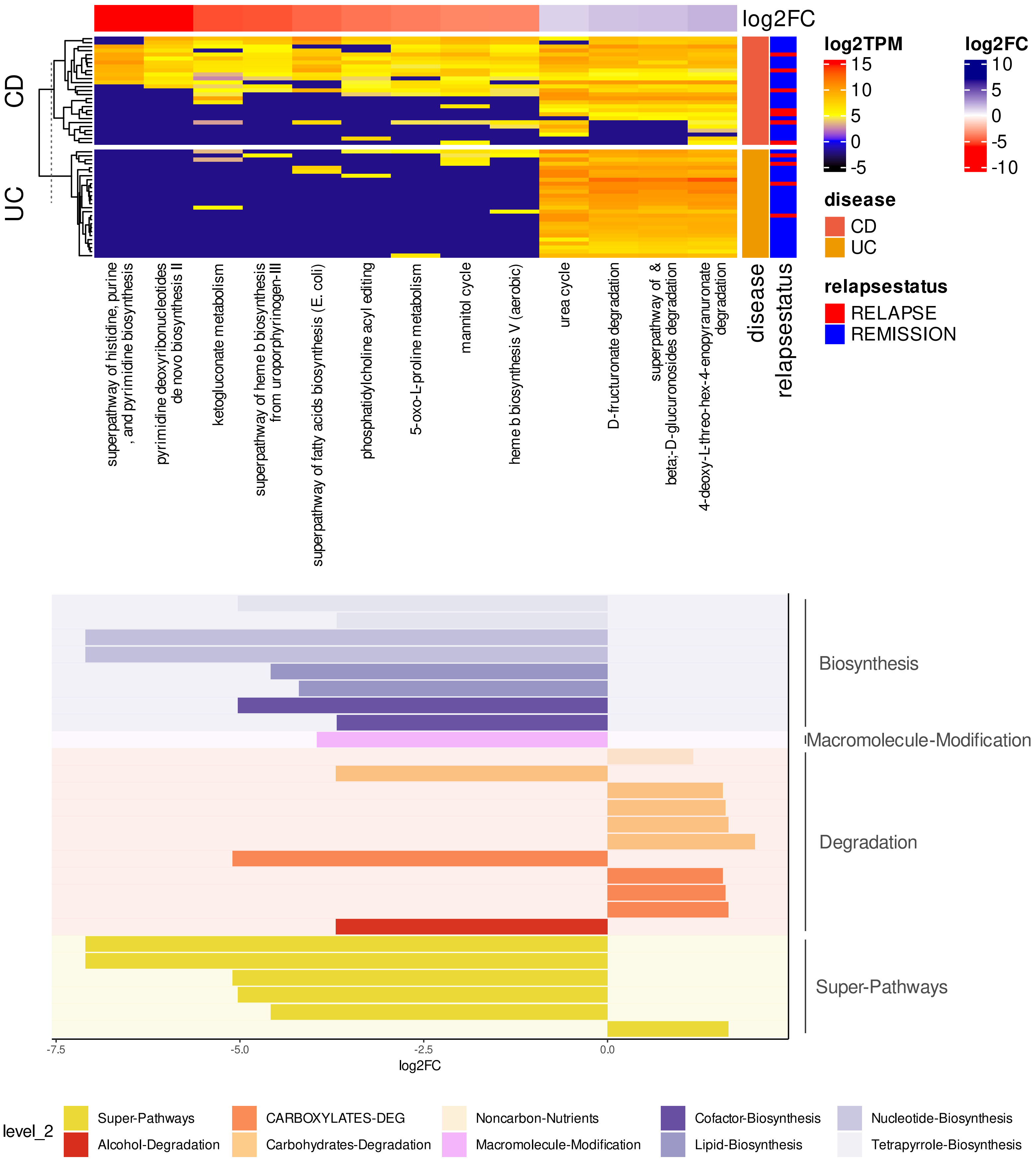

log2FC
CD
UC
log2TPM
15
10
5
0
-5
log2FC
10
5
0
-5
-10
disease
CD
UC
relapsestatus
RELAPSE
REMISSION
superpathway of histidine, purine , and pyrimidine biosynthesis
pyrimidine deoxyribonucleotides de novo biosynthesis II
ketogluconate metabolism
superpathway of heme b biosynthesis from uroporphyrinogen-III
superpathway of fatty acids biosynthesis (E. coli)
phosphatidylcholine acyl editing
5-oxo-L-proline metabolism
mannitol cycle
heme b biosynthesis V (aerobic)
urea cycle
D-fructuronate degradation
superpathway of & beta;-D-glucuronosides degradation
4-deoxy-L-threo-hex-4-enopyranuronate degradation
disease
relapsestatus
Biosynthesis
Macromolecule-Modification
Degradation
Super-Pathways
-7.5
-5.0
-2.5
0.0
log2FC
level_2
Super-Pathways
Alcohol-Degradation
CARBOXYLATES-DEG
Carbohydrates-Degradation
Noncarbon-Nutrients
Macromolecule-Modification
Cofactor-Biosynthesis
Lipid-Biosynthesis
Nucleotide-Biosynthesis
Tetrapyrrole-Biosynthesis

Supplementary Figure 4. Heatmap of the log2-transformed normalized abundance of the enriched and depleted pathways in the microbiome of CD patients (n=27) compared to UC patients (n=27) (A). Differentially expressed pathways in the microbiome of CD patients compared to UC patients (B). Pathways were grouped according to their level 1 MetaCyc classes (broader functionality) and coloured by their level 2 MetaCyc classes (more specific). CD-depleted pathways are represented with positive log2FC, while CD-enriched pathways are negative. Differentially expressed pathways were obtained using mixed-effects linear models.

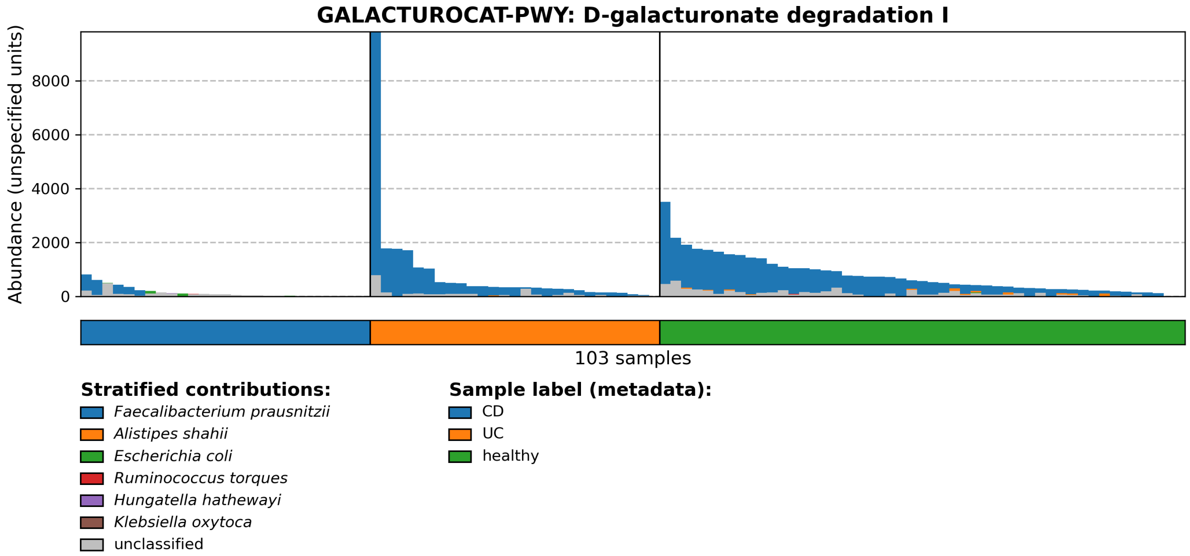
GALACTUROCAT-PWY: D-galacturonate degradation I
Abundance (unspecified units)
0
2000
4000
6000
8000
103 samples
Stratified contributions:
Faecalibacterium prausnitzii
Alistipes shahii
Escherichia coli
Ruminococcus torques
Hungatella hathewayi
Klebsiella oxytoca
unclassified
Sample label (metadata):
CD
UC
healthy

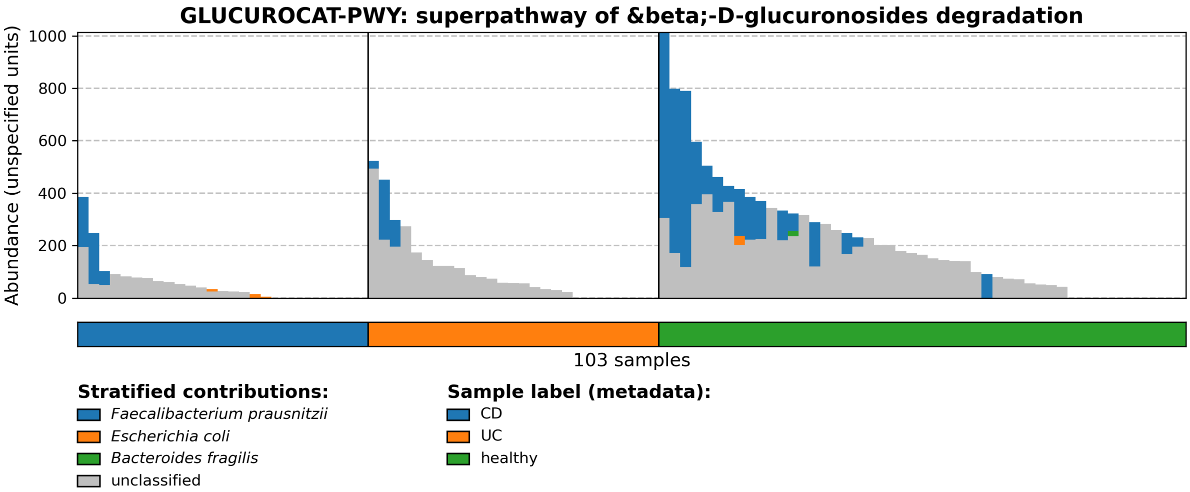
GLUCUROCAT-PWY: superpathway of β-D-glucuronosides degradation
Abundance (unspecified units)
0
200
400
600
800
1000
103 samples
Stratified contributions:
Faecalibacterium prausnitzii
Escherichia coli
Bacteroides fragilis
unclassified
Sample label (metadata):
CD
UC
healthy

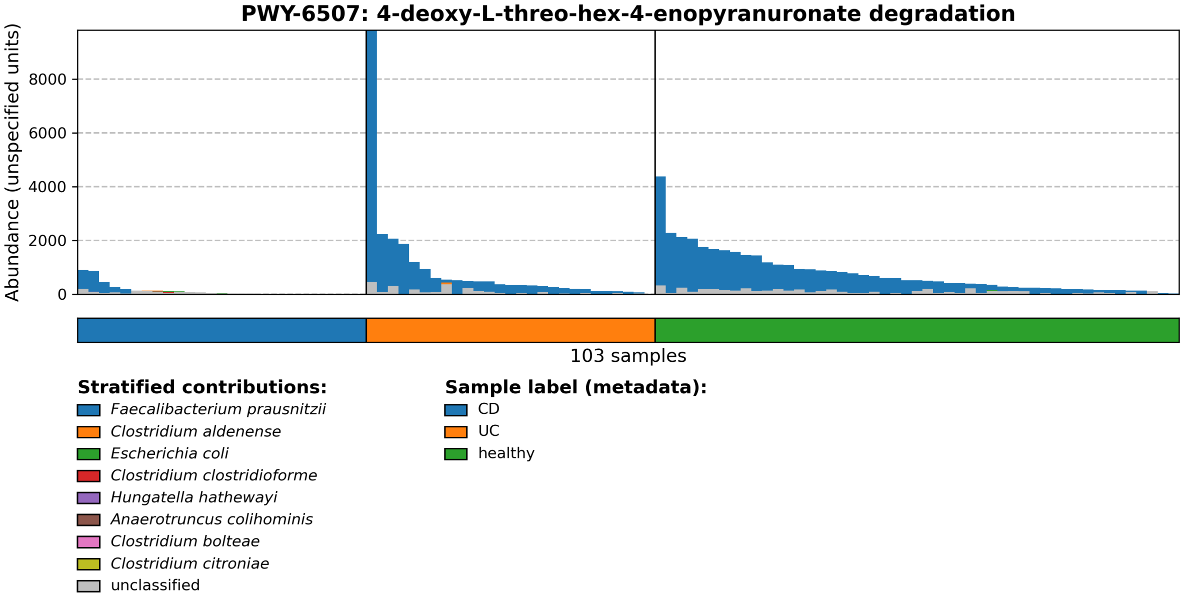
PWY-6507: 4-deoxy-L-threo-hex-4-enopyranuronate degradation
Abundance (unspecified units)
0
2000
4000
6000
8000
103 samples
Stratified contributions:
Faecalibacterium prausnitzii
Clostridium aldenense
Escherichia coli
Clostridium clostridioforme
Hungatella hathewayi
Anaerotruncus colihominis
Clostridium bolteae
Clostridium citroniae
unclassified
Sample label (metadata):
CD
UC
healthy

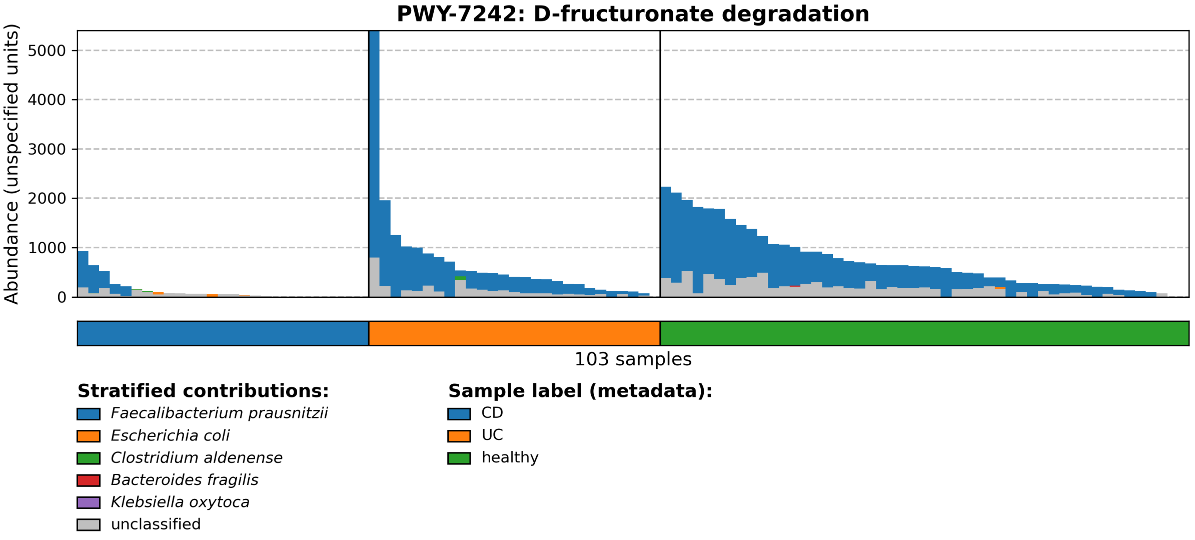
PWY-7242: D-fructuronate degradation
Abundance (unspecified units)
0
1000
2000
3000
4000
5000
103 samples
Stratified contributions:
Faecalibacterium prausnitzii
Escherichia coli
Clostridium aldenense
Bacteroides fragilis
Klebsiella oxytoca
unclassified
Sample label (metadata):
CD
UC
healthy

Supplementary Figure 5. Species contribution to several carbohydrate degradation pathways in CD, UC and HC (CD n=27, HC n=49, UC n=27).

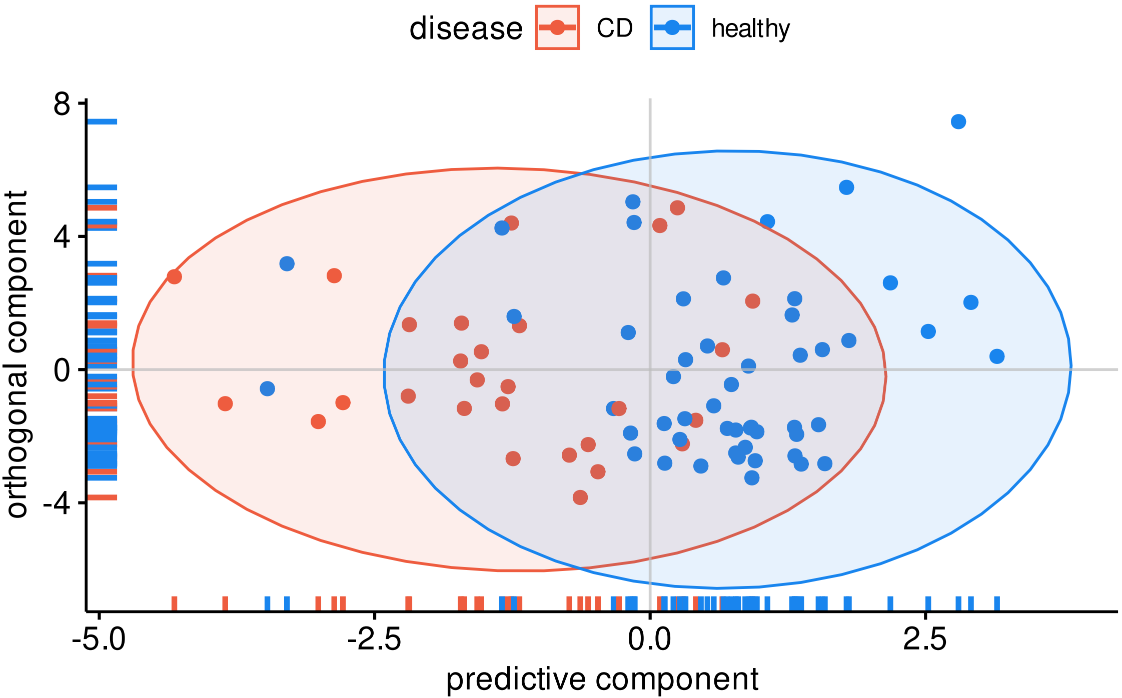

disease
CD
healthy
orthogonal component
8
4
0
-4
-5.0
-2.5
0.0
2.5
predictive component

Supplementary Figure 6. Score plot of the OPLS-DA model between CD (n=28) and HC (n=51) (7-fold CV, 100 permutations, R2Y(cum) = 0.39, Q2Y(cum) = 0.19, pR2Y = 0.01, pQ2Y = 0.01).

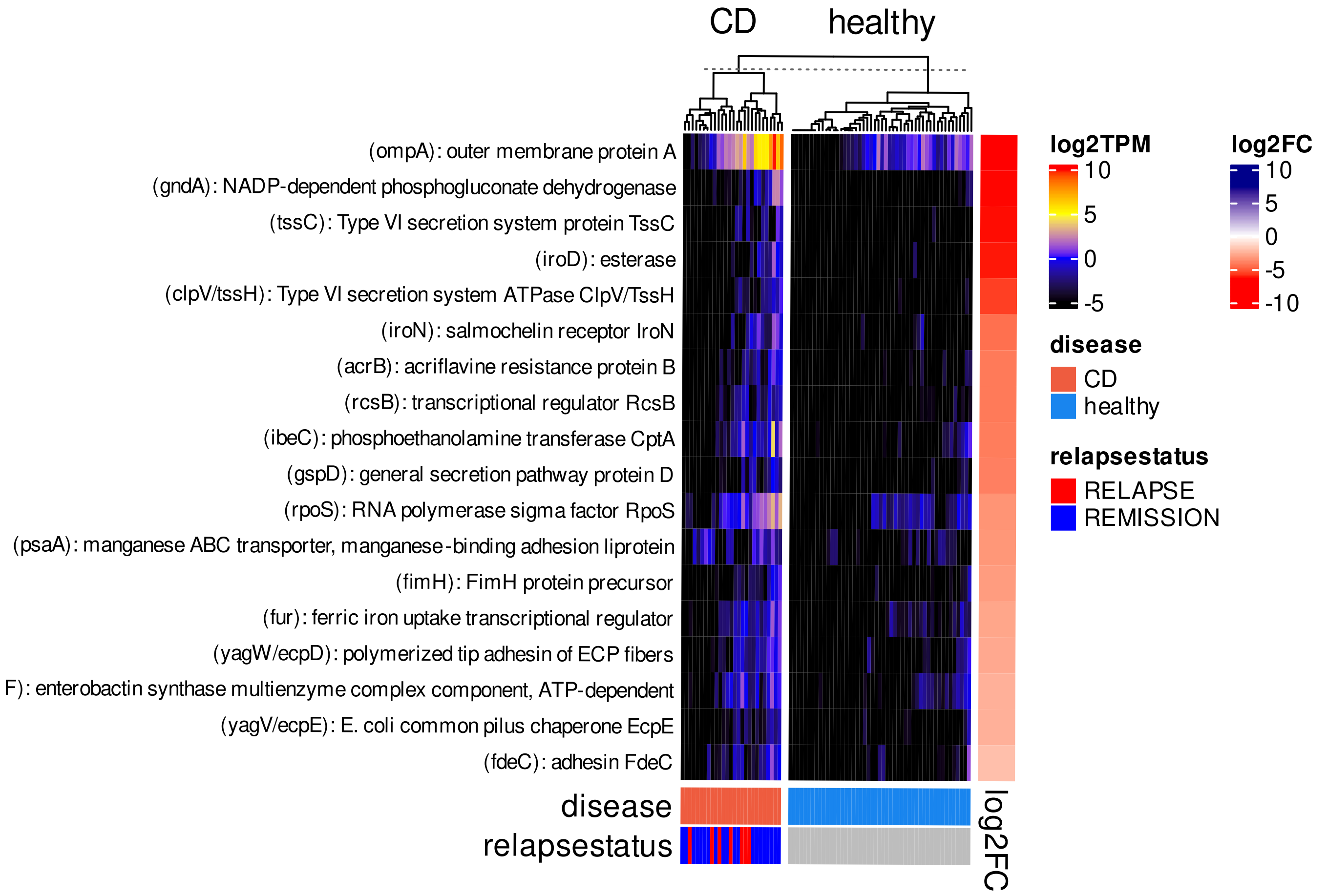
CD
healthy
(ompA): outer membrane protein A
(gndA): NADP-dependent phosphogluconate dehydrogenase
(tssC): Type VI secretion system protein TssC
(iroD): esterase
(clpV/tssH): Type VI secretion system ATPase ClpV/TssH
(iroN): salmochelin receptor IroN
(acrB): acriflavine resistance protein B
(rcsB): transcriptional regulator RcsB
(ibeC): phosphoethanolamine transferase CptA
(gspD): general secretion pathway protein D
(rpoS): RNA polymerase sigma factor RpoS
(psaA): manganese ABC transporter, manganese-binding adhesion liprotein
(fimH): FimH protein precursor
(fur): ferric iron uptake transcriptional regulator
(yagW/ecpD): polymerized tip adhesin of ECP fibers
F): enterobactin synthase multienzyme complex component, ATP-dependent
(yagV/ecpE): E. coli common pilus chaperone EcpE
(fdeC): adhesin FdeC
disease
relapsestatus
log2FC
log2TPM
10
5
0
-5
log2FC
10
5
0
-5
-10
disease
CD
healthy
relapsestatus
RELAPSE
REMISSION

Supplementary Figure 7. Heatmap of the log2-transformed normalized abundance of the enriched virulence factor genes (VF)in the microbiome of CD patients (n=27) compared to healthy controls (n=49).
